# Swallowing and quality of life after total laryngectomy and pharyngolaryngectomy

**DOI:** 10.1016/S1808-8694(15)30496-1

**Published:** 2015-10-19

**Authors:** Dèbora dos Santos Queija, Juliana Godoy Portas, Rogèrio Aparecido Dedivitis, Carlos Neutzling Lehn, Ana Paula Brandão Barros

**Affiliations:** 1 MSc in Sciences - Graduate Program in Health Sciences - Hospital Heliòpolis - HOSPHEL, São Paulo, Brazil. Specialist in voice - CEV, São Paulo. Speech and Hearing Therapist - Voice, Speech and Swallowing Rehab Center - Heliòpolis Hospital - HOSPHEL, São Paulo, Brazil; 2 Specialist in voice - CEV, São Paulo. Speech and Hearing Therapist - Voice, Speech and Swallowing Rehab Center - Heliòpolis Hospital - HOSPHEL, São Paulo, Brazil; 3 Associate Professor - Fundação Lusìada UNILUS, Professor at the Graduate Program in Health Sciences Heliòpolis Hospital, São Paulo; 4 Medicine - Graduate Program in Otolaryngology and Head and Neck Surgery - São Paulo Federal University - Paulista School of Medicine, São Paulo, Brazil, Chair of the Head and Neck Surgery Program - Hospital Heliòpolis, São Paulo, Brazil; 5 PhD in Oncology - Fundação Antônio Prudente, FAP, São Paulo, Brazil, Head of the Voice, Speech and Swallowing Center - Heliòpolis Hospital - HOSPHEL, São Paulo, Brazil. Hospital Heliòpolis - São Paulo

**Keywords:** deglutition, laryngectomy, laryngeal neoplasms, quality of life, deglutition disorders

## Abstract

Dysphagia can be a consequence of total laryngectomy even in the absence of symptoms and it could indeed directly or indirectly compromise quality of life.

**Aim:**

To evaluate the characteristics of swallowing after total laryngectomy and pharyngolaryngectomy with pharyngeal T closure, correlating them with the Quality of Life in Swallowing Disorders questionnaire.

**Methods:**

A prospective evaluation was performed in 28 patients; fifteen undergoing total laryngectomy and thirteen undergoing total pharyngolaryngectomy. Swallowing was evaluated through videofluoroscopy regarding the preparatory, oral and pharyngeal phases of swallowing, and the quality of life related to swallowing questionnaire was employed to measure quality of life.

**Results:**

Anatomical and functional changes were observed under videofluoroscopic evaluation. Dysphagia was diagnosed in 18 patients (64.3%), being mild in 66.6% and moderate/severe in 33.3%. The questionnaire indicated good quality of life in almost all scales. Complaints of dysphagia were associated to the burden (p=0.036) and mental health scale (p=0.031). The questionnaire indicated impact on the mental health scale for patients with severe dysphagia (p=0.012).

**Conclusions:**

High incidence of dysphagia was observed in some quality of life assessments, especially of mild degree.

## INTRODUCTION

Treatment for advanced laryngeal and hypopharyngeal cancer, being surgical or not, can cause anatomical, functional and psychosocial sequelae^1^. Surgery aims at curing and keeping the organ's function which are determined by tumor extension, the amount of tissue remaining and the reconstruction technique. In tumors in which there is the need to remove the entire larynx or in cases of hypopharyngeal tumors with the need to remove the larynx, closing the remaining larynx, in most of the cases done primarily, can be performed in two ways, cross-sectional or in T^2^.

In hypopharynx tumors, especially those originating at the pyriform recess, part of the pharyngeal wall is resected together with the primary tumor that stems from it. Thus, the pharyngeal remains is smaller, thus one should use the “T closure” technique or the flap^3^.

However, anatomical changes can cause dysphagia in 10% to 58% of the patients^4^,^5^. The technique used to close is considered a factor associated to the presence of anatomical changes^6^. In a study assessing 28 patients submitted to total laryngectomy (TL), 11 of them with vertical closure and 17 with the T closure, observed the presence of an anterior pocket in 67% of the patients with T closure and in all the patients submitted to vertical closure^6^.

In 1962, a study of the pharynx after TL through videofluoroscopic exam of the pharynx and esophageal pressure suggests that the dysphagia in these patients is associated to tumor recurrence, stenosis, pharyngeal regurgitation, pocket development or dyscoordination of constricting muscles contraction^7^.

The swallowing manofluoroscopy exam (simultaneous manometry and fluoroscopy) was used with the intent of checking the very dynamics of the oral and pharyngeal phases of patients submitted to TL and pharyngolaryngectomy (PL). During the assessment we observed an increase in oral transit time in the entire sample. The increase in laryngeal transit time was also another alteration found in all the patients. The authors mention that different factors, such an inadequate tongue movement, the loss in the upper and lower hypopharynx and the presence of an anterior pocket interfere with swallowing after TL and PL. They stress that the incidence of dysphagia in these patients can be underestimated due to the lack of symptoms^8^.

Some symptoms, such as regurgitation, the feeling of food build up and prolonged meal time can also indicate the presence of an anterior pocket^4^. The fact is that most patients do not complain and symptoms are usually underappreciated^4^, ^5^, ^6^, ^7^, ^8^, ^9^, ^10^, ^11^, ^2^, ^13^, ^14^, ^15^, ^16^. Many of these patients use feeding adaptations which most of the times imply feeding restrictions^17^.

When discussing swallowing in these patients, we do not know how much of it is functional and whether or not feeding restrictions impact their quality of life (QL). The impact inherent to treatment sequelae is a factor to be considered not only from the clinical and/or surgical view point, but also from the patient's view point, since the concept of QL has specific issues associated with psychosocial factors^18^.

Many studies have recently discussed QL after head and neck cancer treatment by means of generic or specific questionnaires. However, swallowing is approached as one more aspect which makes it difficult to interpret its importance in QL. The UW-QOL (University of Washington Quality of Life) questionnaire, recently validated in Brazil, proved to be more sensitive regarding the aspects associated with swallowing; however, not clearly identifying its interference in QL^19^, ^20^. It is necessary to include specific domains which go more in-depth regarding aspects associated with swallowing and its impact on QL^21^, ^22^.

McHorney et al. created the SWAL-QOL (Quality of life in swallowing disorders) questionnaire - a specific tool which assesses the impact of swallowing alterations in QL^23^, ^24^, ^25^. Barros et al. used the SWAL-QOL questionnaire to investigate QL associated with swallowing after surgical treatment for advanced laryngeal cancer and identified the impact of swallowing alterations in food selection, social function and desire scales. The scores were higher for those patients who reported diets with food consistence restriction^26^. In the literature we did not find papers associated with swallowing functional evaluation with the specific QL after TL and PL.

The goal of the present investigation was to analyze swallowing characteristics and QL associated with swallowing (SWAL-QOL) after TL and PL with primary T closure.

## MATERIALS AND METHODS

For the prospective study we surveyed the data base available at the Head and Neck Ward of the Institution, looking for surgeries performed between 1996 and 2006. Participants in this study were those patients with histopathological diagnosis of squamous cell carcinoma, clinically staged by the criteria established by the International Union for Cancer Fighting (IUCF)^27^ and submitted to PL and TL, with primary closure in T. As inclusion criterion we considered those patients submitted to TL with or without partial pharyngectomy for curative purposes alone or associated with radiotherapy, through the neck-facial areas added or not to the area of supraclavicular fossa. We excluded the patients submitted to other surgical treatments in the head and neck region and with neurological alterations, because of their interference on the physiology of swallowing.

We selected 138 charts, and from these, 58 patients had recurrence, metastasis or had died. It was not possible to contact 52 of the patients. The sample was made up of 28 patients with average age of 58.8 years. The study was approved by the Ethics in Research Committee of our institution under protocol # 484.

We studied the personal information, swallowing complaints, the stomatognathic system and swallowing by means of swallowing videofluoroscopy and the SWAL-QOL questionnaire^23^, ^24^, ^25^.

Swallowing videofluoroscopy was carried out in all the patients using the Philips radiological equipment (Philips Chalanger®, N 800 HF), carried out by one radiologist and one speech therapist. The patients remained seated in the anteroposterior and lateral positions during the exam and the device's image focus was previously defined by the lips anteriorly, the hard palate superiorly, the posterior neopharynx posteriorly and inferiorly by the airway bifurcation and the esophagus. The patients were instructed to swallow liquid (water and barium at a 1:1 ratio) and solid substances mixed to barium. 5ml (spoon) and 20ml (cup) of liquid were given in a continuous swallowing. For solids, the patients were instructed to chew the cookie before swallowing. We analyzed the oropharyngeal motility and the presence of stasis and the dysphagia severity according to Zerbinatti^11^.

The preparatory and oral phases were analyzed according to formation, control and ejection of the chewed food; the movement of the tongue against the palate and antero-posterior. These variables were considered adequate and inadequate, and when inadequate, the alteration was classified in relation to the degree as mild, moderate or severe. Oral cavity stasis was also analyzed as present or absent, and when present they were classified as mild moderate or severe. The pharyngeal phase was analyzed taking into account the tongue contact against the pharynx, the presence of anatomical alterations, such as a pocket on the anterior wall, cricopharyngeal mobility, and stasis in the anterior and posterior walls of the neopharynx. In order to better standardize the alterations presented during videofluoroscopic evaluation, we used the protocol adapted for TL and PL. The degree of dysphagia was classified according to a four-point scale^11^ (Chart 1).

**Chart 1:** Dysphagia classification scale^11^.

Dysphagia degree classification		Characteristics
D0	Normal swallowing	Without stasis or food consistency restriction.
D1	Mild dysphagia	With mild stasis, without food consistency restriction.
D2	Moderate dysphagia	With mild/moderate stasis, there can be restrictions of up to two consistencies.
D3	Severe dysphagia	Moderate to severe stasis, with the restriction of more than one food consistencies.

In order to analyze the results, judgment was divided in the pharyngeal and preparatory oral phases. The analysis of subjective judgment qualitative variables was carried out in a consensus by 3 speech therapists with more than 5 years of experience in interpreting post TL and PL swallowing videofluoroscopy functional analysis.

The SWAL-QOL questionnaire was used after translation and cultural adaptation for Brazilian Portuguese following internationally accepted guidelines. The questionnaire is made up of 44 questions distributed in 10 scales: burden, desire, symptom frequencies, food selection, communication, fear, mental health, social function, sleepiness and fatigue. The questions regarding the scales approach some aspects which associate the items aforementioned to swallowing, such as, for instance, in the burden scale: “It is very difficult to deal with my swallowing problem …” in the fear scale: “I am afraid of developing pneumonia”; in the sleep scale: “Do you have any problems when sleeping?” The answers were converted into scores that vary between 0 and 100 (worse and best scoring) 23, 24, 25. The questionnaire also assesses the patient's self-perception as far as his global health is concerned.

The description of variables served the following measures-summary: parametric variables: frequency (minimum, maximum, simple mathematical average, standard deviation, median and respective totals, when the case being) and non-parametrical variables: frequency and percentage. The Mann-Whitney test was applied to check the differences between the two categories of base-variables: the Kruskal-Wallis test to check the differences among three or more base-variable categories and the chi-squared test, adjusted by the Fisher's Statistics, in order to verify the degree of association among the pairs of variables of interest.

## RESULTS

We had 28 patients participating in this study, 15 submitted to TL and 13 to PL, with mean age of 58.8 years. Clinical, demographic and treatment characteristics are specified on Table 1.

**Table 1:** Demographic, clinical and treatment characteristics. (n=28)

Variable	Category	Freq. (%)
	Min.-max.	42-82
Age (years)	Median	57
	Mean±sd	58,8±10,3
Gender	Males	26 (92,8)
	Females	2 (7)
Tumor site	Larynx	15 (53,5)
	Pharynx+larynx	13 (46,5)
T	3	17 (60,7)
	4	11 (39,3)
N	0	22 (78,5)
	+	6 (21,4)
M	0	28 (100)
	+	0
Surgery	Total larnyngectomy Pharyngolaryngectomy	15 (53,5)
		13 (46,5)
Reconstruction	T Closure T	28 (100)
Radiotherapy	No	3 (10,7)
	Yes	25 (89,3)
	Min.-max.	5000-7200
Radiotherapy dose	Median	6300
	Mean±sd	6486±547,38
Chemotherapy	No	24 (85,7)
	Yes	4 (14,3)
Time to end of treatment (months)	Min.-max.	1-84
	Median	24
	Mean±sd	31,9±26,5
Postoperative complications	Pharyngocutaneous fistula	5 (17,8)
	Granuloma in the stoma	2 (7)
	Oral ingestion without restrictions for solid food	17 (60,7)
Current feeding	Oral ingestion with restrictions for solid food	8 (28,5)
	Exclusive alternative pathway	3 (10,7)

Legend: Freq.: frequency; min. -minimum; max. -maximum; sd. - standard deviation.

At the time of assessment, three patients (10.7%) used other alternative feeding sources, two used a nasogastric tube and one had a gastrostomy. The patient being fed through a gastrostomy tube had been previously submitted to laryngeal cancer treatment by radiotherapy (organ-sparing protocol) and had severe alterations in tongue mobility and was starting treatment in order to adapt and to learn strategies to facilitate the swallowing oral phase and guarantee nutritional support. Two patients were fed by nasogastric tube because of a pharyngocutaneous fistula.

In the interview, 6 (21.4%) patients reported swallowing complaints, and 3 of them (10.7%) had TL and 3 (10.7%) PL.

In assessing the stomatognathic system, we noticed that 21 (75%) patients were partially edentulous and 7 (25%) were total edentulous. Tongue mobility alterations were found in 2 patients, one of congenital origin and the other was the one aforementioned - submitted to the organ-sparing treatment.

In doing a videofluoroscopic evaluation of swallowing, especially the oral preparatory phase, there was a predominance of swallowing food formation alteration in 12 exams (42.8%) followed by an increase in oral transit time in 10 (35.7%) of the cases. In the pharyngeal phase we identified the prevalence of stasis in the oropharynx in 18 (64.2%) tests and in the hypopharynx in 12 (48.3%). Another alteration seen was reflux, 2 (7%) were esophageal and 1 (3.5%) pharyngo-oral. As far as anatomical alterations are concerned, we identified anterior wall pocket, cricopharyngeal bar and anastomosis stenosis. Swallowing was considered functional in 10 (35.7%) cases and dysphagia was diagnosed in 18 (64.3%) patients. Mild degree dysphagia (D1) was seen in 12 (43%) of the tests, moderate (D2) in 4 (14%) and severe (D3) in 2 (7%). In order to facilitate the statistical analysis, we redistributed the sample and grouped the cases classified with moderate to severe dysphagia (D2 / D3). Thus, the degree of dysphagia was redistributed in D1 and D2 / D3. The dysphagia severity grade distribution is shown on Table 2.

**Table 2:** Videofluoroscopic evaluation of swallowing. (n=28)

Variable	Category	Freq. (%)
Oral-preparatory phase		
Chewed food clustering	Adequate	16 (57)
	Inadequate	12 (42,8)
Oral transit time	Adequado	18 (64,3)
	Inadequado	10 (35,7)
Oral cavity stasis	Absent	20 (71,5)
	Present	8 (28,5)
Pharyngeal phase		
Tongue contact × pharynx	Adequate	21 (75)
	Inadequate	7 (25)
Oropharyngeal stasis	Absent	10 (35,7)
	Present	18 (64,3)
Pharyngeal mobility	Adequate	17 (60,7)
	Inadequate	11 (39,3)
Hypopharyngeal stasis	Absent	16 (57)
	Present	12 (43)
Pharyngeal transit time	Adequate	17 (60,7)
	Inadequate	11 (39,3)
Anterior wall pocket	Absent	23 (82)
	Present	5 (18)
Cricopharyngeal bar	Absent	22 (78,5)
	Present	6 (21,5)
Anastomosis stenosis	Absent	27 (96,5)
	Present	1 (3,5)
Esophageal reflux	Absent	26 (93)
	Present	2 (7)
Pharyngo-oral reflux	Absent	27 (96,5)
	Present	1 (3,5)
Tongue mobility alteration	Absent	26 (93)
	Present	2 (7)
Dysphagia	No	10 (35,7)
	Yes	18 (64,3)
	D0	10 (35,7)
Classification	D1	12 (42,8)
	D2/D3	6 (21,4)

Legend: Freq.: frequency; D0: Functional swallowing; D1: Mild dysphagia; D2/D3: Moderate/severe dysphagia.

As to the degree of alterations, both in the oral-preparatory phase as well as in the pharyngeal phase we observed a prevalence of the mild degree in relation to all the variables analyzed.

The scores achieved in the SWAL-QOL questionnaire indicated good QL. The communication (47.7), feeding duration (50.3) and social function (66) scales had lower scores. Score distribution regarding each scale is specified on Table 3.

**Table 3:** Quality of life associated with swallowing - SWAL-QOL. (n=28)

Variable	Min.-max.	Median	Mean±sd
Burden	0-100	81	77,6±26
Feeding duration	0-100	50,3	50,3±39,5
Desire	0-100	83,3	70,2±31,8
Symptom frequency	32,7-98	77	77,3±17
Food selection	0-100	87,5	75,4±28,5
Communication	0-100	50	47,7±32,4
Fear	12,5-100	78	76±24,7
Mental health	6,25-100	100	78,8±30
Social function	0-100	77,5	66±37,3
Sleep	50-100	75	79±17,3
Fatigue	25-100	79	74,3±24

Legend: SWAL-QOL - Quality of Life in Swallowing Disorders; min.: minimum; ma×.:ma×imum; sd.:standard deviation.

Health was classified as excellent by 4 (14%) patients in the group, very good by 8 (29%), good by 13 (46.4%) and regular by 3 (10.7%). In order to facilitate statistical analysis, we grouped the patients who considered their health excellent with the group with very good health. Thus, the self-classification stratification of health was distributed as excellent/very good: 12 (42.8%), good: 13 (46.4%) and regular: 3 (10.7%).

The stratified analysis of complaints and the SWAL-QOL indicated a greater QL impact associated with swallowing in those patients who reported swallowing difficulties. The Mann-Whitney test indicated a score difference in the two groups, with statistical significance for the patients who complained of their swallowing in the scales of burden (p=0.036) and mental health (p=0.031). We also noticed lower scores in the group with swallowing complaints; however, without statistical significance in the feeding duration (29±40), social function (40±41.5), communication (41.6±28), desire (68.3±28.7) and fear (67.7±32.2) scales.

As we compare the degree of dysphagia and QL associated with swallowing, the scores point to a greater impact on the feeding duration scale with a greater involvement associated with severe dysphagia - D2 / D3 (29±40). In this group, the social function (40±41.5), communication (41.6±28), burden (58.3±33-2) and desire (61±28.7) scales showed lower scores, without statistical meaning. We noticed a difference among these three groups (DOX D1 X D2 / D3) with statistical significance considering the mental health scale (p=0.030). (Table 4)

**Table 4:** Association between the SWAL-QOL and the presence or absence of a diagnosis of dysphagia. (n=28)

Variable	Category	Dysphagia				
		D0(n = 10)	D1 (n = 12)	D2 / D3 (n=6)	Total (n=28)	P
Burden	Mèdia±dp	86,2±19,9	80,2±23,5	58,3±33,2	77,3±16,9	0,925
Feeding duration	Mèdia±dp	51,2±40,5	60±37,5	29±40	50,3±39,5	0,271
Desire	Mèdia±dp	85,9±19,2	61,7±38	61±28,7	70,2±31,7	0,143
Frequency of symptoms	Mèdia±dp	79,7±14,9	76,8±17,4	74±21,5	77,3±16,9	0,938
Food selection	Mèdia±dp	77,5±28,7	76±32	70,8±24,5	75,4±28,5	0,870
Communication	Mèdia±dp	51±37,4	47,9±32,3	41,6±28	47,7±32,4	0,806
Fear	Mèdia±dp	82,5±19,7	74,5±25	67,.7±32,2	75,9±24,6	0,574
Mental health	Mèdia±dp	95±15,8	78,7±26,7	52±39,2	78,8±30,2	0,030*
Social function	Mèdia±dp	82,5±23,8	65,4±39,4	40±41,5	66±37,3	0,123
Sleep	Mèdia±dp	77,5±21	83,3±12,2	72,9±20	79±17,3	0,541
Fatigue	Mèdia±dp	71,5±23,9	70,8±24	86±23,9	74,3±23,9	0,434

Legend: SWAL-QOL - Quality of Life in Swallowing Disorders; min.: minimum; ma×.:ma×imum; sd.:standard deviation; p value obtained by the Kruskall-Wallis p <0.05. * -statistical significance.

Since we observed a statistically significance difference in the mental health scale of the SWAL-QOL questionnaire, we applied the Mann-Whitney test in order to pin-point the differences among the dysphagia grade categories. There was a difference between D0 and D2 / D3 (p=0.012).

As we analyzed the characteristics of swallowing alterations according to surgery type (TL X PL), we noticed a predominance of alterations in the pharyngeal and preparatory-oral phases in the group submitted to PL in almost all the variables analyzed. However, the chi-squared test adjusted by the Fisher's statistics indicated a statistically significant difference for the inadequate oral transit time in the group submitted to PL (p=0.002).

As far as food consistency goes, we have noticed that 30.7% of the patients submitted to PL and 26.3% to TL had restrictions to solid food. We noticed exclusive alternative pathway feeding in 15.3% of the PL group and in 6.7% from the TL. When we applied the Fisher Adjusted Chi-Squared test to check the association among pairs of variables, we did not observe statistically significant differences (p=0.602).

Dysphagia was detected in the two groups – 10 (66.7%) in the TL and 11 (84.6%) in the PL. In the 2 groups, dysphagia was predominantly mild (TL – 46.7%, PL −61.5%).

As we checked the SWAL-QOL scores in the two surgical groups - TL and PL, we observed lower scores in the two groups in the feeding duration (TL– 51.6±42.4; PL-48.7±37.4) and communication (TL– 55±29.8; PL– 39.4±34.5) scales. When we compared the scores, we observed a greater difference in the food selection (TL– 80.8±23.5; PL-69.2±33.3) and social function (TL– 80.6±28. PL– 49.2±40.5) scales. However, these differences were not statistically significant.

## DISCUSSION

In the interview, only a small percentage (21.4%) complained of swallowing. These patients were fed either by an alternative pathway or with consistency restrictions. However, the food consistency adaptation was reported by 28.5% of the sample. The dysphagia symptom in these patients may manifest because of the effects of radiotherapy in the oropharyngeal mobility, due to the absence or lack of dental elements and the adaptation of medium or lower constricting muscles^6^, ^7^, ^8^, ^9^, ^10^, ^11^, ^12^. In this sample, 50% of the subjects who complained of dysphagia were edentulous and they were all submitted to radiotherapy. It is very likely that the alterations caused by the lack of teeth and xerostomia and their respective interference in the oral phase are much more sensitive to the patient's perception than the pharyngeal phase alterations and, because of that, the patient is asymptomatic. Another fact that has to be considered and which can also be an indicator of dysphagia is the difficulty in keep a diet varying food consistencies^14^, ^17^. The particularity of the dysphagia characteristics of these patients seems underappreciated by the clinician and the patient.

Even with the small number of patients with dysphagia, in the videofluoroscopic assessment of swallowing we observed functional and anatomical alterations in the preparatory-oral and pharyngeal phases and dysphagia diagnosed in 64.3% of the tests.

In the oral-preparatory phase we identified especially an inadequate chewed food creation and increased food transit time. Dysphagia can manifest because of the effects of radiotherapy on oropharyngeal mobility, because of the lack of teeth and the very pressure adaptation of medium and lower constricting muscles^6^, ^7^, ^9^, ^12^. The effects of radiotherapy, such as taste alterations, lower saliva production and muscle fibrosis associated to the lack of dental elements can interfere in the oral-preparatory phase and compromise it. In this sample, 50% of the subjects reported swallowing difficulties were edentulous and they had all been submitted to radiotherapy. Only three patients in our study had not been submitted to radiotherapy; however, we did not observe differences between these patients and the rest of the sample.

Dysfunctions of the oral-preparatory phase can interfere in the performance of the subsequent phase by the very relation of continuity of swallowing biomechanical events causing stasis in the mouth and hypopharynx, as seen in the present sample.

Another alteration which can contribute to pharyngeal phase dysfunctions is the anterior wall pocket. Some patients have symptomatic/asymptomatic stasis in the pocket region, regurgitation and difficulties to clear these residues and, consequently, increase in pharyngeal transit time^7^, ^8^, ^10^, ^11^, ^12^. However, it is important to consider that the mere presence of anatomical alteration does not indicate dysfunction^8^. In our study, of the 5 patients with anterior wall pocket, a total of 4 (75%) had dysphagia.

The lack of coordination caused by the adaptation of neopharynx constricting muscles after TL has also been regarded in many studies as the cause for dysphagia. McConnel et al. showed that after TL, the average pharyngeal transit time doubles and there is the need for a strong thrust force in order to overcome pharyngeal resistance^8^. In videofluoroscopic evaluation, inadequate pharyngeal movement and the longer pharyngeal transit time were seen in 39% of the tests. When we compared patients in terms of surgery, we observed a greater occurrence of alterations in those submitted to PL in almost all the variables analyzed, however without statistically significant differences. One study about esophageal mobility of 15 patients after TL found that the upper sphincter of these patients had a significant reduction in resting pressure and of maximal contraction in relaxation extension and movement coordination. The authors point out that the loss caused to the pharyngeal constrictor muscle and the rupture of the pharyngeal plexus, which innervates the pharyngeal constrictor muscle and the cricopharyngeal are responsible for pressure changes after surgery^28^. Because of the very extension of surgery, we expect to have a greater occurrence of swallowing disorders after PL; however, in the sample studied we did not observe differences between the two groups submitted to surgery. It is likely that the reduced sample impacted these findings.

Although dysphagia has been identified in more than half of the samples, in general the SWAL-QOL yielded good scores. These results corroborate the literature an can be justified by the fact that it is likely that patients prioritize disease cure and maybe underappreciate some of its secondary aspects, such as swallowing^18^, ^22^, ^26^. Some studies state that within a period of 6 to 12 months of post-treatment, the scores return or are still higher than those found in the pre-treatment^29^, ^30^. In our study, the patients evaluated had, in average, 31.9 months of post-treatment.

The questionnaire scores indicated specific QL impact for those patients who reported swallowing complaints in the burden and mental health scales. In this same group, the scores were also lower in the during feeding and social function scales, however without statistically significant differences. Severe dysphagia also indicated mental health involvement. Nonetheless, in those cases of functional swallowing, the SWAL-QOL scores were higher, indicating good QL. In those cases of mild and moderate/severe dysphagia, the scores were lower in the feeding duration, desire and social function scales. This data points to the fact that even when mild, dysphagia post TL and PL already represents a problem associated with these factors. The SWAL-QOL seems to have reflected this relationship between functional alterations and QL impact after TL and PL. This fact reinforces the statement that the inclusion of specific domains is an important factor to assess swallowing changes impact on QL^21^, ^22^.

Although both the videofluoroscopic exam and the questionnaire did not indicate differences regarding the results of the two surgical groups, the scores were lower in the PL group considering the feeding duration, social function and food selection scales. It is likely that the surgical extension sequelae be in fact more easily noticeable in patients with social function repercussion. Ward et al noticed in their sample that patients submitted to PL with persistent dysphagia had high levels of disability and disadvantage with repercussions in their social and emotional functions^14^. In general, our results allow us to infer that swallowing alterations after TL and PL can have an association with QL associated with swallowing in a more direct way regarding burden and mental health; and indirect regarding feeding duration and social function.

Some aspects such as the investigation of complaints and diet style can serve as important indicators of the need for an objective assessment such as videofluoroscopy in swallowing. The SWAL-QOL also seems to be a tool that can guide the speech therapist through the patient's perceptions characterizing swallowing alterations after TL.

## CONCLUSIONS

Swallowing after TL and PL with primary closure in a T fashion can be characterized by anatomical and functional alterations. These alterations can cause predominantly mild dysphagia with QL repercussion associated with swallowing.

QL analysis through the SWAL-QOL pointed to an between the presence of moderate/severe dysphagia and QL impact on the mental health scale.

## References

[1] List MA, Ritter-Sterr CA, Lansky SB (1990). A performance status scale for head and neck cancer patients. Cancer..

[2] Qureshi SS, Chaturvedi P, Pai PS, Chaukar DA, Deshpande MS, Pathak KA (2005). A prospective study of pharyngocutaneous fistulas following total laryngectomy. J Cancer Res Ther..

[3] Dedivitis RA, Ribeiro KC, Castro MA, Nascimento PC (2007). Pharyngocutaneous fistula following total laryngectomy. Acta Otorhinolaryngol Ital..

[4] Balfe DM, Koehler RE, Setzen M, Weyman PJ, Baron RL, Ogura JH (1982). Barium Examination of the Esopahgus after Total laryngectomy. Radiology..

[5] Nayar RC, Sharma VP, Arora MML (1984). A study of pharynx after laryngectomy. J Laryngol Otol..

[6] Davis RK, Vincent ME, Shapsay SM, Strong MS (1982). The anatomy of “T” versus vertical closure of the hypopharynx after laryngectomy. Laryngoscope..

[7] Kirchner JA, Scatliff JH, Dey FL, Sheedd DP (1963). The pharynx after laryngectomy. Laryngoscope..

[8] Mc Connel FMS, Mendelsonh MS, Logemann JA (1986). Examination of swallowing after total laryngectomy using manofluorography. Head Neck..

[9] Hui YDLO, Wei WI, Yuen PW, Lam LK, Ho WK (1996). Primary closure of pharyngeal remnant after total laryngectomy and partial pharyngectomy: how much residual mucosa is sufficient?. Laryngoscope..

[10] Pennings RJE, van den Hoogen FJA, Marres HAM (1999). Laser treatment of symptomatic anterior pharyngeal pouches after laryngectomy. Head Neck..

[11] Zerbinatti FAB (2004). Avaliação videofluoroscópica da deglutição após laringectomia e faringolaringectomia total [Monografia de conclusão do curso de pós-graduação Lato Sensu].

[12] Salgado P, Ramos T (2006). Avaliação videofluoroscópica da deglutição após laringectomia total e faringolaringectomia [Monografia de conclusão do curso de pós-graduação Lato Sensu].

[13] Witt ME (1999). Food for life: management of swallowing related issues in head neck cancer. Dev Support Cancer Care..

[14] Ward EC, Bishop B, Frisby J, Stevens M (2002). Swallowing outcomes following laryngectomy and pharyngolaryngectomy. Arch Otolaryngol Head Neck Surg..

[15] Rosales Solis AA, Hernández-Guerrero A, Sobrino Cossio S, Frias Medivil M, Córdov Pluma VH, Granados Garcia M (2004). Pharyngoesophageal stenosis following surgery and radiotherapy in patients with advanced laryngeal cancer. Rev Gastroenterol Mex..

[16] Coleman JJ, Searles JM, Hester TR, Nahai F, Zubowicz V, McConnel FM (1987). Ten years experience with the free jejunal autograft. Am J Surg..

[17] Pillon J, Gonçalves MI, De Biase NG (2004). Changes in eating habits following total and frontolateral laryngectomy. São Paulo Med J..

[18] Mohide EA, Archibald SD, Tew M, Young JE, Haines T (1992). Postlaryngectomy Quality-of-life Dimensions Identified by patients and health care professionals. Am J Surg..

[19] Hassan SJ, Weymuller EAJR (1993). Assessment of quality of life in head and neck cancer patients. Head Neck..

[20] Vartanian JG, Carvalho AL, Yueh B, Priante AV, de Melo RL, Correia LM (2004). Long Term quality of life evaluation after head and neck cancer treatment in a developing country. Arch Otolaryngol Head Neck..

[21] Gliklich RE, Goldsmith TA, Funk GF (1994). Are head and neck specific quality of life measures necessary?. Head Neck..

[22] Mowry SE, LoTempio MM, Sadeghi A, Wang KH, Wang MB (2006). Quality of life outcomes in laryngeal and oropharyngeal cancer patients after chemoradiation. Otolaryngol Head Neck Surg..

[23] McHorney CA, Bricker DE, Kramer AE, Rosenbek JC, Robbins J, Chignell KA (2000). The SWAL-QOL outcomes tool for oropharyngeal dysphagia in adults: I - conceptual foundation and item development. Dysphagia..

[24] McHorney CA, Bricker DE, Robbins J, Kramer AE, Rosenbek JC, Chignell KA (2000). The SWAL-QOL outcomes tool for oropharyngeal dysphagia in adults: II - item reduction and preliminary scaling. Dysphagia..

[25] McHorney CA, Robbins J, Lomax K, Rosenbek JC, Chignell K, Kramer AE (2002). The SWAL-QOL and SWAL-CARE outcome tools for oropharyngeal dysphagia in adults: III - documentation of reliability and validity. Dysphagia..

[26] Barros APB, Portas JG, Queija DS, Lehn CN, Dedivitis RA (2007). Autopercepção da desvantagem vocal (VHI) e qualidade de vida relacionada à deglutição (SWAL-QOL) de pacientes laringectomizados totais. Rev Bras Cir Cabeça Pescoço..

[27] Sobin LH (1997). TNM Classification of malignant tumours: larynx.

[28] Choi EC, Hong WP, Kim CB, Yoon CH, Nam JI, Son EJ (2003). Changes of esophageal motility after total laryngectomy. Otolaryngol Head Neck Surg..

[29] Terrell JE, Ronis DL, Fowler KE, Bradford CR, Chepeha DB, Prince ME (2004). Clinical predictors of quality of life in patients with and neck cancer. Arch Otolaryngol Head Neck Surg..

[30] Murry T, Madasu R, Martin A, Robbins KT (1998). Acute and chronic changes in swallowing and quality of life following intraarterial chemoradiation for organ preservation in patients with advanced head and neck cancer. Head Neck..

